# Supplementary material for: Comparison of the Basal Cell Carcinoma (BCC) Tumour Microenvironment to Other Solid Malignancies
Source: Cancers (Basel). 2023 Jan 2;15(1):305. doi: 10.3390/cancers15010305 (PMC9818508; doi:10.3390/cancers15010305)
Supplement: Supplementary file 1 [file cancers-15-00305-s001.zip › cancers-2102069-supplementary.pdf]

**Supplementary Table S1.** Summary of all significant and top three (if available, in bold) closest BCC "relatives" by cell type.

| Cell Type | BCC score (median), Q1, Q3 | BCC- "Relatives" score (median) | Q1 | Q3 | P-value |
|---|---|---|---|---|---|
| Th2 | 4.188E-18, 0, 0.01980129 | **PRAD = 1.07E-17** | **0** | **0.01706565** | **0.6102** |
| | | **KICH = 0.001056705** | **0** | **0.007381045** | **0.151** |
| | | **KIRC = 2.23E-17** | **0** | **0.03051409** | **0.1368** |
| MSC | 0.188433346, 0.08336783, 0.28205374 | **SKCM = 0.16096867** | **0.07202425** | **0.25446426** | **0.3872** |
| Total CD8+ | 0.00445073, 0, 0.043739228 | **GBM = 0.01048155** | **0** | **0.03021879** | **0.2741** |
| | | **LGG = 0** | **0** | **0.01996548** | **0.05719** |
| Tgd | 0, 0, 0.008231755 | **THCA = 0** | **0** | **0.008063912** | **0.7752** |
| | | **CHOL = 0** | **0** | **0.01282003** | **0.6099** |
| | | **LIHC = 0** | **0** | **0.006720286** | **0.5979** |
| | | KICH = 0 | 0 | 0.009986598 | 0.5525 |
| | | TGCT = 0 | 0 | 0.003538575 | 0.3495 |
| | | GBM = 0 | 0 | 0.001140639 | 0.244 |
| | | KIRC = 0 | 0 | 0.002117059 | 0.2002 |
| | | DLBC = 0 | 0 | 0.01388940 | 0.08334 |
| | | SARC = 0 | 0 | 0.002129824 | 0.07291 |
| | | UCEC = 0 | 0 | 0 | 0.05902 |
| B-cells | 0.04857795, 0.020757612, 0.086950544 | **THYM = 0.055183104** | **0.03108108** | **0.08455359** | **0.3715** |
| | | **GBM = 0.04815724** | **0.028318876** | **0.075344991** | **0.2374** |
| Total Lymphocytes | 0.39140159, 0.3132104, 0.4688854 | **SARC = 0.416998654** | **0.3124302** | **0.5153758** | **0.2290** |
| Macrophages | 0.25033106, 0.1763854, 0.3784326 | **ESCA = 0.2331247** | **0.1675734** | **0.3352133** | **0.5397** |
| | | **KICH = 0.276729** | **0.1843325** | **0.3949821** | **0.4904** |
| | | **BLCA = 0.2590653** | **0.16693727** | **0.33762817** | **0.3333** |
| | | ACC = 0.2594566 | 0.2071582 | 0.3565666 | 0.3128 |
| | | STAD = 0.2654186 | 0.19623848 | 0.33075342 | 0.2366 |
| | | HNSC = 0.2748354 | 0.2069556 | 0.3561280 | 0.2252 |
| | | DLBC = 0.2793964 | 0.18870890 | 0.33007984 | 0.08976 |
| Total NK | 0.0325587, 0.005046464, 0.053041073 | **THYM = 0.02682497** | **0.009408674** | **0.049124771** | **0.6696** |
| | | **ESCA = 0.02523973** | **0.01152386** | **0.04604726** | **0.4214** |
| | | **KIRP = 0.02878749** | **0.01382830** | **0.04874396** | **0.3381** |
| | | UVM = 0.02839242 | 0.01406619 | 0.04997250 | 0.25 |
| | | READ = 0.02042911 | 0.003387114 | 0.041380355 | 0.2457 |
| | | UCS = 0.0360689 | 0.01366458 | 0.07025842 | 0.2434 |
| | | KIRC = 0.03447636 | 0.01423560 | 0.05709336 | 0.2311 |
| | | SARC = 0.02980665 | 0.01438558 | 0.04549609 | 0.1979 |
| | | BLCA = 0.03280019 | 0.01570095 | 0.05698518 | 0.1718 |
| | | DLBC = 0.01879405 | 0.004128464 | 0.042910416 | 0.1221 |

| | | | | | |
|---|---|---|---|---|---|
| | | PRAD = 0.03551566 | 0.01577075 | 0.05472112 | 0.09082 |
| | | LUSC = 0.02415559 | 0.01062867 | 0.03874510 | 0.07355 |
| | | CHOL = 0.04473911 | 0.02815367 | 0.06960667 | 0.05944 |
| | | COAD = 0.02242313 | 0.006309632 | 0.041589770 | 0.05544 |
| | | THCA = 0.03297953 | 0.01616638 | 0.05686914 | 0.0512 |
| | | SKCM = 0.02038228 | 0.006983169 | 0.040457560 | 0.05057 |
| Tregs | 0, 0, 0 | **ACC = 0** | **0** | **0** | **0.9991** |
| | | **LAML = 0** | **0** | **0.00315034** | **0.3533** |
| Total CD4+ | 0.15296126, 0.1031139, 0.2082608 | **CHOL = 0.156189** | **0.1321129** | **0.1952696** | **0.6959** |
| | | **SARC = 0.1562586** | **0.1154291** | **0.2083701** | **0.6334** |
| | | **SKCM = 1412604** | **0.08728384** | **0.19168763** | **0.5715** |
| | | UCS = 0.145859 | 0.08257441 | 0.22042976 | 0.5447 |
| | | THCA = 0.1411155 | 0.1003041 | 0.1867856 | 0.4649 |
| | | LIHC = 0.1652248 | 0.1152202 | 0.2237863 | 0.4642 |
| | | BLCA = 0.1649314 | 0.09478596 | 0.23077402 | 0.2715 |
| | | MESO = 0.1472571 | 0.11229869 | 0.18824632 | 0.2135 |
| | | CESC = 0.1667974 | 0.1167771 | 0.2087164 | 0.2112 |
| | | KICH = 0.1782413 | 0.12870279 | 0.22144281 | 0.17 |
| | | PAAD = 0.1700216 | 0.12820877 | 0.21796094 | 0.1254 |
| | | TGCT = 0.1730064 | 0.1346265 | 0.2090150 | 0.08706 |
| | | PCPG = 0.175342 | 0.13049092 | 0.23039868 | 0.08318 |
